# Differences in Type I Interferon Signaling Antagonism by Dengue Viruses in Human and Non-Human Primate Cell Lines

**DOI:** 10.1371/journal.pntd.0003468

**Published:** 2015-03-13

**Authors:** Freddy A. Medina, Giselle Torres-Malavé, Amanda J. Chase, Gilberto A. Santiago, Juan F. Medina, Luis M. Santiago, Jorge L. Muñoz-Jordán

**Affiliations:** 1 Centers for Disease Control and Prevention, Division of Vector-Borne Diseases, Dengue Branch, San Juan, Puerto Rico, United States of America; 2 University of Puerto Rico Medical Science Campus, Department of Microbiology & Medical Zoology, San Juan, Puerto Rico, United States of America; 3 Mercer University School of Medicine, Division of Basic Medical Sciences, Macon, Georgia, United States of America; University of North Carolina at Chapel Hill, UNITED STATES

## Abstract

**Background/Objectives:**

In vitro studies have shown that dengue virus (DENV) can thwart the actions of interferon (IFN)-α/β and prevent the development of an antiviral state in infected cells. Clinical studies looking at gene expression in patients with severe dengue show a reduced expression of interferon stimulated genes compared to patients with dengue fever. Interestingly, there are conflicting reports as to the ability of DENV or other flaviviruses to inhibit IFN-α/β signaling.

**Methodology/Principal Findings:**

In order to determine the relative inhibition of IFN-α/β signaling by DENVs, a method combining flow cytometry and a four-parameter logistic regression model was established. A representative isolate from DENV-1, -3 and -4 and seventeen representative isolates encompassing all DENV-2 genotypes were evaluated. All of the DENVs evaluated in this study were capable of inhibiting IFN-α/β signaling. Most of the strains were able to inhibit IFN-α/β to a degree similar to DENV strain 16681; however, DENV-2 sylvatic strains demonstrated an increased inhibition of phosphorylated signal transducer and activator of transcription (pSTAT1). Surprisingly, we were unable to observe inhibition of pSTAT1 by DENV-2 sylvatic strains or the Asian strain 16681 in non-human primate (NHP) cell lines. Analysis in primary *Rhesus macaque* dendritic cells suggests that DENVs are capable of inhibiting IFN signaling in these cells. However, contrary to human dendritic cells, production of IFN-α was detected in the supernatant of DENV-infected *Rhesus macaque* dendritic cells.

**Conclusions:**

The ability of DENVs to inhibit IFN-α/β signaling is conserved. Although some variation in the inhibition was observed, the moderate differences may be difficult to correlate with clinical outcomes. DENVs were unable to inhibit pSTAT1 in NHP cell lines, but their ability to inhibit pSTAT1 in primary *Rhesus macaque* dendritic cells suggests that this may be a cell specific phenomena or due to the transformed nature of the cell lines.

## Author Summary

Dengue is a viral illness acquired through the bite of an infected mosquito. This flu-like illness, which in rare instances can be fatal, threatens more than half of the world’s population. Both *in vitro* and clinical studies looking at how the virus operates have consistently found that the interferon response is modulated by the virus during infection. We looked at the ability of dengue virus (DENV) strains to inhibit phosphorylated signal transducer and activator of transcription (pSTAT1) after IFN-β stimulation and observed that contrary to earlier published reports; all DENVs are capable of inhibiting IFN-α/β signaling. Strains from the DENV-2 sylvatic genotype, which mainly infect non-human primates (NHP), displayed an increased ability to inhibit pSTAT1 compared to the Asian strain 16681. To our surprise, DENVs were only capable of inhibiting pSTAT1 in human cell lines, but not in NHP cell lines. Inhibition of pSTAT1 is observed in both human and NHP primary dendritic cells. These results have important implications in the use of NHP cell lines for studies of IFN-α/β inhibition by DENV *in vitro* and may be a relevant consideration when using NHPs for DENV pre-clinical studies.

## Introduction

More than half of the world’s population is at risk of acquiring an acute mosquito-borne illness known as dengue [1]. Infected individuals can be asymptomatic or display a range of clinical features. Many symptomatic dengue patients experience a mild fever, however, some develop severe dengue complications resulting in plasma leakage, hemorrhage, and organ impairment [2].

Dengue virus (DENV) contains a ∼10.7 kb positive strand RNA genome that encodes 3 virus structural proteins (C, prM, and E) and seven nonstructural (NS) proteins (NS1, 2A, 2B, 3, 4A, 4B and 5) [3]. There are four serotypes of DENV (DENV-1, -2, -3, & -4) and each is further sub-classified into genotypes. Some studies have observed differences in virological characteristics and clinical outcomes that associate with certain genotypes [4–7]. So far, these correlates of disease severity have been most extensively studied in the DENV-2 genotypes. The key elements hypothesized to contribute to disease outcome come from both virus molecular determinants and host factors [5,8–10].

The acute nature of DENV infections suggests that the innate immune system plays a vital role in its elimination. Type I interferon (IFN-α/β) is produced in response to the detection of DENV RNA by various pathogen-recognition receptors [11,12]. The IFN-α/β produced can bind cell surface receptors and cause dimerization of the IFN-α/β receptor subunits [13]. As a result, the JAK/STAT pathway is activated. The phosphorylation of STAT1 creates binding sites that allow homodimerization of STAT1 and heterodimerization of STAT1–2 [14,15]. STAT1 or STAT1–2 dimers are joined with IRF9/p48 to form a trimeric complex named ISGF3 [16,17]. The mature ISGF3 complex functions as a transcription factor that enters the nucleus and binds to promoter sequences in DNA containing interferon stimulated response elements. Over three hundred interferon stimulated genes are induced by IFN-α/β signaling [18]. These genes encode products that help uninfected cells to establish an antiviral state [19–22].

DENV has evolved to thwart the IFN-α/β antiviral response. Initial studies demonstrated that established DENV infections *in vitro* were refractory to the inhibitory effects of IFN-α/β on replication [23]. Gene array studies of clinical samples from DENV infected individuals have highlighted the relevance of the interferon system with regards to pathogenic outcomes. These studies have shown that the interferon system contains some of the most highly regulated genes during infection. Patients with severe dengue, such as dengue hemorrhagic fever or dengue shock syndrome, show suppression of IFN-α/β-stimulated genes compared to those with dengue fever [24,25].

Pathologically relevant flaviviruses such as JEV, WN, KUN, and TBE also inhibit the signal transduction cascade exerted by IFN-α/β [26–29]. Some studies have suggested that not all DENV or flaviviruses are capable of blocking IFN-α/β signaling [30]. Furthermore, studies of JEV and WNV strains have suggested a correlation between disease severity and the ability to inhibit IFN-α/β signaling [31–33]. Our aim in this study was to determine if these observations could be extended to DENV. However, the variable plaque morphology and growth characteristics make it difficult to compare the differential effect flaviviruses may exert on host signaling pathways. In this study we developed a quantitative method that allowed us to determine the relative IFN-α/β blocking ability among DENV strains. Comparisons were made with low-passage clinical isolates from all DENV serotypes and with representative DENV-2 strains of the American, Asian, American/Asian, cosmopolitan, and sylvatic genotypes. In contrast to previous observations with DENV and other flaviviruses, we show that all DENVs are capable of blocking IFN-α/β signaling. Most strains suppressed pSTAT1 to levels similar to those observed with DENV strain 16681, except for the DENV2 sylvatic genotype. The degree to which DENVs block IFN-α/β signaling did not correlate with their replication. The correlation of the strength of IFN-α/β signaling inhibition and pathogenic potential will likely be difficult to demonstrate due to the modest variations observed between DENV strains.

In this study we show that there are differences in inhibition of pSTAT1 by DENVs in human and non-human primate (NHP) cell lines. However, DENVs are capable of inhibiting pSTAT1 in both human and Rhesus macaque primary dendritic cells. Previous studies with human dendritic cells showed that DENVs inhibit IFN-α/β production. Our results show that Rhesus macaque primary dendritic cells readily produce IFN-α when infected. This is the first study to suggest possible differences in the innate immune response to DENVs in humans and NHPs.

## Materials and Methods

### Cell lines and dengue virus isolates

*Aedes albopictus* C6/36 cells (ATCC #CRL-1660, Manassas, VA) and A549 human lung epithelial cells (ATCC # CCL-185, Manassas, VA) were cultivated in DMEM (Invitrogen, Grand Island, NY) supplemented with 10% heat-inactivated fetal bovine serum (FBS), L-glutamine, and nonessential amino acids. C6/36 cells were maintained at 33°C and A549 cells at 37°C with 5% CO2. Vero African green monkey cells (ATCC # CCL-81, Manassas, VA) were cultivated in M199 medium (Invitrogen, Grand Island, NY) supplemented with 10% heat-inactivated FBS L-glutamine and nonessential amino acids and maintained at 37°C.

Twenty DENV strains were used in this study. Four low-passage strains representing each of the DENV serotypes (DENV1: 101–001/PR1998, DENV2: BID-V681, DENV3: BID-V1610, and DENV4: BID-V2442) were obtained from the Centers for Disease Control Dengue Branch Passive Dengue Surveillance System (San Juan, Puerto Rico) [34]. For studies using DENV-2 genotypes, representative strains TH/DB052/2003, 203–001/TW1987, 201–001/PR2006, and BID-V585 were obtained from the Centers for Disease Control Dengue Branch Passive Dengue Surveillance System (San Juan, Puerto Rico); DENV-2 strains 16681, and PR-159 were obtained from the Centers for Disease Control dengue reference strain collection; DENV-2 strains 131, IQT2133, Ven2, K0049, Mara3, 1349, and ArA6894 were kindly provided by Dr. R. Rico-Hesse (Baylor College of Medicine, Houston, TX); DENV-2 strains DakAr510, DakAr75505, and DkD811 were kindly provided by Dr. Nikos Vasilakis (University of Texas Medical Branch, Galveston, TX).

### Plaque assay

Plaque assays were performed as described previously [35]. Images of stained plaques were taken in a Bio-Rad Molecular Imager Gel Doc XR+ System (Bio-Rad, Hercules, CA). The area (mm ± standard deviation) of each plaque was determined by means of an image analyzing program (Digimizer® version 4.2.1, MedCalc Software, Mariakerke, Belgium).

### Preparation of DENV isolates and titration

DENV stocks were titered by flow cytometry using a previously described method that yields results similar to the standard plaque assay [36]. Infected cells were harvested, fixed with BD Cytofix, permeabilized with BD Cytoperm (BD Biosciences, San Jose, CA) and stained with the Alexa 647 conjugated monoclonal antibody (MAb) 2H2 (prM-specific, dengue-complex cross-reactive MAb). Unconjugated 2H2 was kindly provided by Dr. Robert Putnak, Walter Reed Army Institute of Research. Cell infectivity was quantified with a BD FACS Calibur and using BD Cell Quest software. The titer of the virus was determined using the following formula: fluorescence-activated cell sorting (FACS) infectious units/ml = [(% of infected cells) × (total number of cells per well) × (dilution factor)]/ (volume of inoculum added to cells).

### Measurement of IFN inhibition by flow cytometry

DENV strains were used to infect A549 cells at an MOI of 2 for 24 hours. Cells were then stimulated for 30 minutes with 500 U/ml IFN-β (PBL interferon source, Piscataway, NJ) and fixed with 2% paraformaldehyde. After methanol permeabilization, cells were co-stained with anti-pSTAT1 Alexa 488- and anti-DENV prM Alexa 647-conjugated antibodies. Cell fluorescence was measured on a BD FACS Calibur and data analysis was conducted using BD Cell Quest software. DENV infected (prM+) cells were gated and analyzed to determine the percentage of pSTAT1(+) cells.

### Determination of the relative inhibition of pSTAT1 by DENV strains

**Fig 1:**
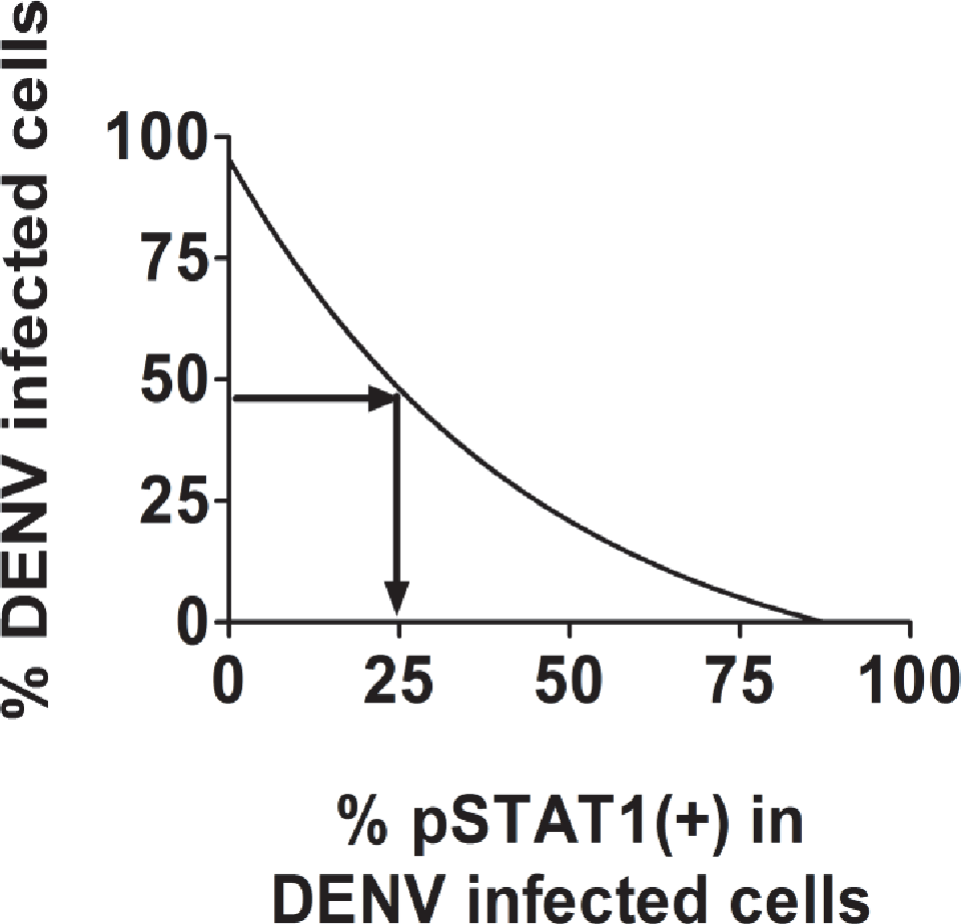
Sample graph for standard curve of percent DENV infected cells vs. percent pSTAT1 positive in DENV infected cells.

**Fig 2:**
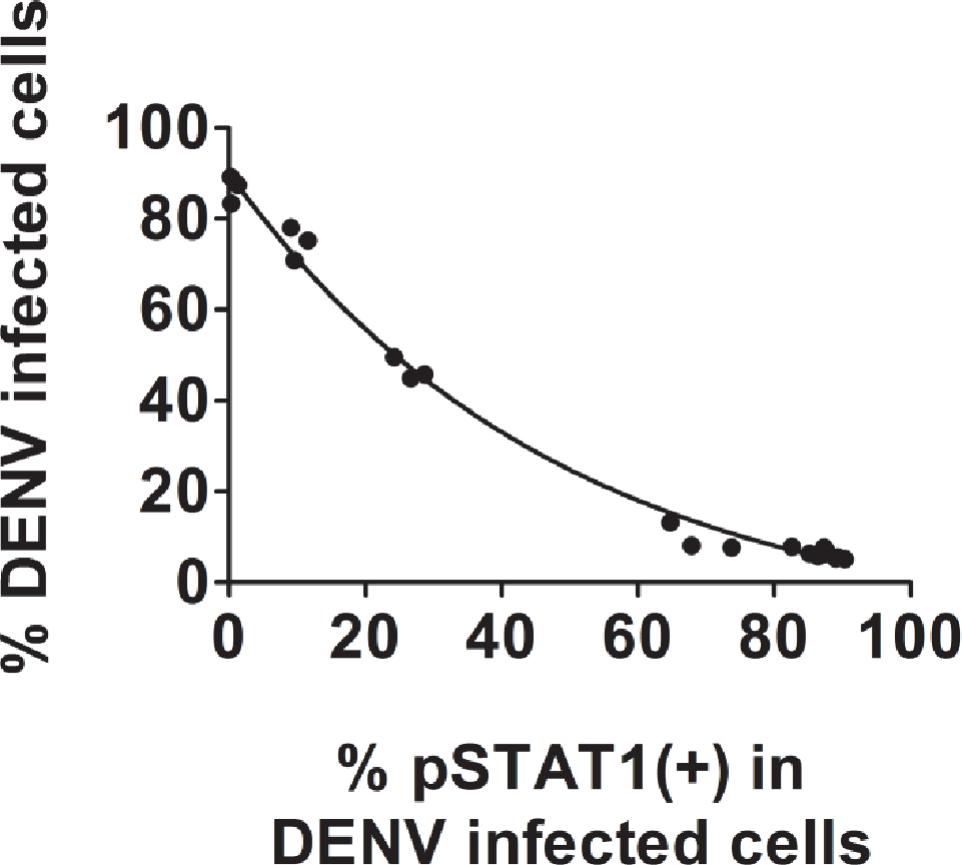
Inverse correlation of pSTAT1 inhibition with DENV infectivity in prM(+) gated cells A549 cells were infected with serial 1:3 dilutions of DENV strain 16681 beginning with an MOI = 6. Twenty-four hours post-infection cells were stimulated for 30 minutes with IFN-β. Cell staining was done using anti-pSTAT1 Alexa 488- and anti-DENV prM Alexa 647-conjugated antibodies. Quantification of cell fluorescence was performed on a FACSCalibur. An analysis gate was placed on the DENV+ population and the percent of pSTAT1+ cells was determined. Experiments were performed in triplicate. Results shown are representative of four independent experiments.

The pSTAT1 inhibition assay is performed akin to an ELISA assay with a standard curve. To construct the graph in Figs. 1 and 2, the percent of cells that stained DENV positive was calculated and a gate was placed on the DENV positive population at each of the virus dilutions to analyze the percent of cells that also stained positive for pSTAT1. The data points for graphs 1 and 2 display as curved shape, therefore, instead of using the formula y = Mx + b to calculate the expected percent of inhibition from DENV strains by linear regression analysis a 4-parameter logistic curve fit was performed to obtain the expected percent inhibition. The expected DENV inhibition of pSTAT1 was calculated using a 4-parameter logistic (4PL) model developed from a standard curve of serially diluted DENV strain 16681 infected cells that were stimulated with IFN-β. Serial 1:3 dilutions of 16681 were performed starting with an MOI = 6. The expected inhibition of pSTAT1 by DENV strains at the obtained infectivity was calculated using the four-parameter logistic regression (4PL) model fit using the following equation:

$$f(x)=c+\frac{d-c}{1+\exp\left(b\left(\log(x)-\log(e)\right)\right)}$$

x = expected % inhibition of pSTAT1

b = slope factor

c = the response at zero infection

d = the response at infinite % infection

e = mid-range inhibition of pSTAT1 by DENV 16681

Using Fig. 1 as a crude example, we extrapolate the data to have a rough estimate of how much inhibition of pSTAT1 would be expected if a strain blocked IFN to the same degree as 16681. For example, if strain A had the same ability to inhibit pSTAT1 as 16681 and approximately 45% of the cells were infected, we would expect to see only 25% of the DENV positive cells to also be pSTAT1 positive (see Fig. 1). In this assay, any strain can be used as the reference strain.

The relative inhibition of pSTAT by the DENV strain was calculated by: (Expected pSTAT1 inhibition by 16681 at the obtained percent of infection; as determined above)–(observed pSTAT1 inhibition by DENV strain).

### DENV replication and DENV quantitative real-time RT-PCR

A549 cells were infected with representative DENV strains at an MOI = 0.1 for 1 hour at 37°C. After removal of the virus inoculum, cells were washed four times with PBS and grown in DMEM containing 2% FBS. Supernatants from infected cells were collected at 12, 24, and 48 hours post-infection. Samples (280 μl) were processed for RNA extraction on an automated M48 BioRobot using the MagAttract Viral RNA Kit (Qiagen, Valencia, CA). The changes in DENV amplification over time were assessed using a standard curve of in-vitro transcribed RNA generated from plasmids containing amplicons for DENV1 (NS5 gene), DENV2 (E gene), DENV3 (M gene), and DENV4 (M gene) using AmpliScribe T7-Flash Transcription Kit (Epicentre Biotechnologies, Madison, WI) per manufacturer's protocol. Contaminating plasmid DNA was removed with the Ambion TURBO DNA-free Kit (Life Technologies, Grand Island, NY) and RNA was quantified using a NanoDrop ND-1000 Spectrophotometer. Starting with 100 μg of in-vitro transcribed RNA, eight serial 1:10 dilutions were performed. We performed real time RT-PCR to quantify DENV genome copy equivalents/ml of the sample using a previously described method[37].

### Western blot

Human (A549, Huh7) and monkey (LLCMK2, Vero) cell lines were infected with DENV-2 16681 at an MOI of 5 for 24 hrs. After a 30 minute stimulation with IFN-β, cell lysates were prepared with RIPA buffer containing a protease inhibitor and phosphatase inhibitor cocktail (Roche Diagnostics GmbH, Mannheim, Germany). Samples were clarified by centrifugation at 12,000 x g for 10 min. at 4⁰C and protein concentrations were quantified using the bicinchoninic acid reagent (Thermo Scientific Pierce, Rockford, IL). The volume required for 10 μg of protein was loaded and separated by sodium dodecyl sulfate-polyacrylamide gel electrophoresis and transferred to nitrocellulose. The primary antibodies in this study were used at the manufacturer’s recommended dilutions: anti-pSTAT1 and anti-GAPDH (Cell Signaling Technologies, Beverly, MA), anti-STAT1 (Sigma-Aldrich, St. Louis, MO), anti-pSTAT2 and anti-STAT2 (R&D Systems, Minneapolis, MN), anti-DENV NS4B (Abcam, Cambridge, MA). Horseradish peroxidase-conjugated secondary antibodies (1:5,000 dilution; Jackson ImmunoResearch, West Grove, PA) were used to visualize bound primary antibodies with the Supersignal chemiluminescence substrate (Thermo Scientific Pierce, Rockford, IL).

### Statistical analysis

DENV strains were compared to 16681 as a control by one-way analysis of variance (ANOVA) followed by Dunnett's Multiple Comparisons Test in GraphPad 5 (GraphPad InStat, GraphPad Software, San Diego, CA). Statistical differences in IFN-α/β inhibition between individual viruses were assessed by unpaired two-tailed T-test analysis. Differences were considered significant when p < 0.05.

### Phylogenetic analysis

The phylogenetic trees of DENV strains utilized in this study were created with Mega 5, using maximum likelihood analysis based on nucleotide sequences of the complete envelope gene.

## Results

### Assessment of the relative inhibition of pSTAT by DENV strains in A549 infected cells

One of our objectives was to compare the ability of DENV strains to inhibit IFN-α/β signaling. To help account for differences in strain infectivity when using the same MOI, we developed a method for quantifying inhibition of IFN-α/β signaling by DENV through inhibition of STAT-1 phosphorylation (pSTAT1). We selected pSTAT1 as a marker of IFN antagonism based on its relatively high expression compared to other molecules in the JAK/STAT pathway and because detection can be readily achieved by flow cytometry with commercially available MAbs. To establish our assay we used the prototypical DENV strain 16681, previously shown to inhibit IFN-α/β stimulation, as our reference virus [38]. The inhibition of IFN signaling was assessed by flow cytometry of A549 cells that were infected with serially diluted 16681 and treated with IFN twenty-four hours post-infection. Only cells that stained positive for DENV M protein were gated and analyzed for STAT1 phosphorylation at each dilution. A decrease in pSTAT1 expression indicated an inhibition of the IFN signaling cascade. Our results show that inhibition of pSTAT1 was observed in all of the virus dilutions. The DENV blockade was still observed at the lowest MOI (0.025) where approximately 10% of the cells were infected. However, a comparison between the different MOIs demonstrates that the magnitude of the inhibition, or percentage of pSTAT1(+) cells, depends on the percentage of infected cells. The percentage of pSTAT1(+) cells was inversely correlated with the percentage of DENV infected cells (Fig. 2 and Table 1). These results suggest that only analyzing DENV(+) cells is insufficient for making comparisons between strains and that the level of infection must be accounted for when determining the relative IFN antagonism potential. In order to normalize for differences in infectivity we created a dose response curve from A549 cells infected with serial dilutions of 16681 and stimulated with IFN-β. The dose-response curve was fit using a 4PL logistic regression and an equation was created from the data sets to obtain the expected percent of pSTAT1(+) cells at any given amount of infection (see Materials and Methods). This allows us to use the 16681 IFN dose-response curve as a reference to which we can compare the relative IFN antagonism of DENV strains.

**Table 1:** Percent inhibition of pSTAT1 in DENV infected A549 cells at different MOIs.

DENV 16681 MOI	Average percent of DENV-infected cells	Average percent of pSTAT1(+) cells in DENV-infected cells
0	0.61	86.69
0.025	10.07	74.69
0.074	26.54	46.73
0.22	68.78	9.66
0.66	85.13	7.13
2	87.04	6.61
6	89.56	5.39

### Representative DENV serotypes and DENV-2 sylvatic genotypes display some variation in their inhibition of IFN-α/β signaling

**Fig 3:**
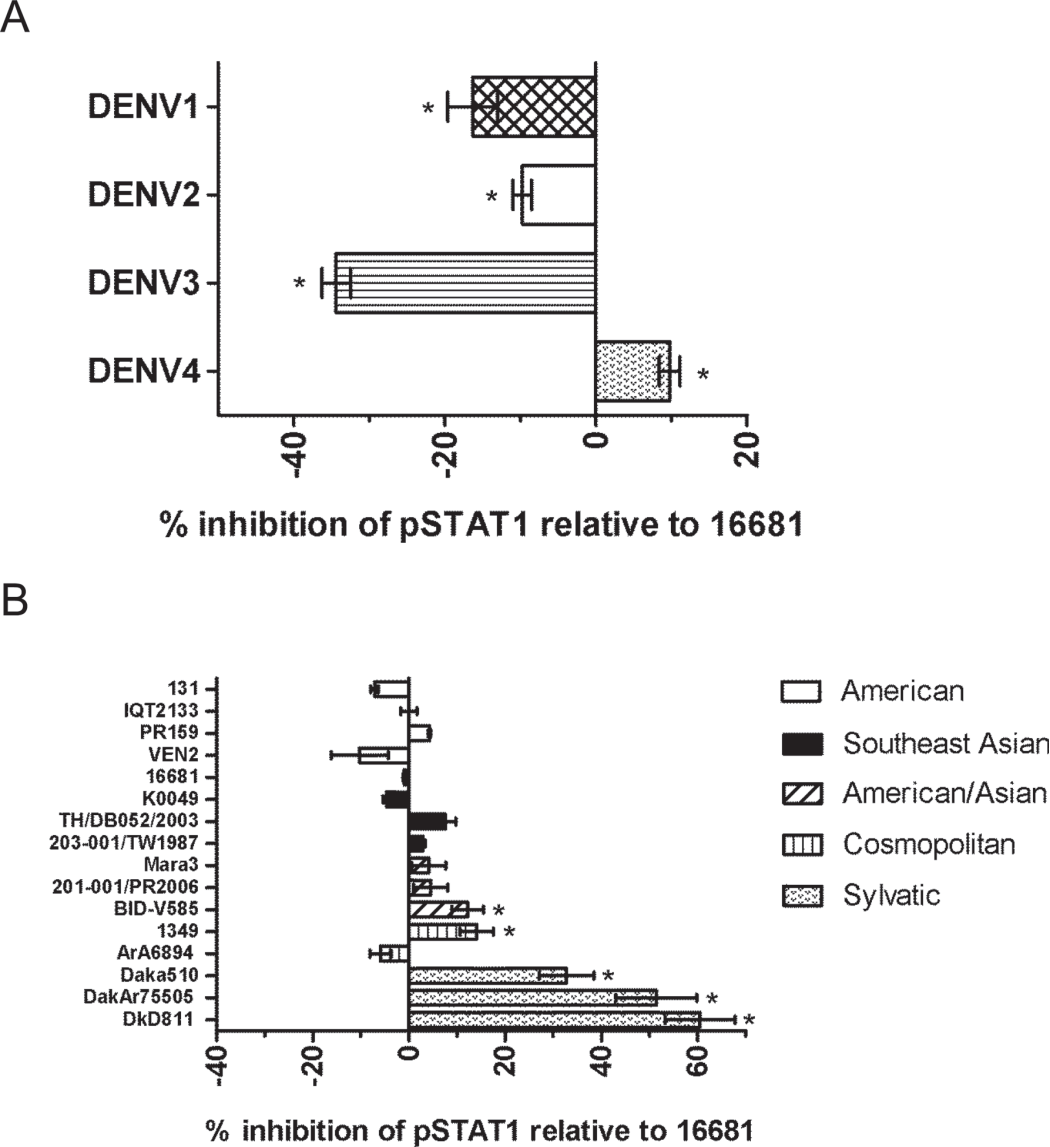
Variable inhibition of pSTAT1 by DENV clinical isolates. (**A**) Clinical isolates from each DENV (1–4) serotype or (**B**) from all DENV-2 genotypes (American, Southeast Asian, Asian/American, and cosmopolitan) were tested for their ability to inhibit IFN-α/β signaling. A549 cells were infected with DENV strains at an MOI = 2 for 24 hours and stimulated with IFN-β for 30 minutes. Cells were processed and stained with fluorescently labeled anti-DENV and anti-pSTAT1 antibodies. The percent of pSTAT1 was calculated from cells in cells infected with DENV. Experiments were performed in triplicate. Results shown are representative of four independent experiments. Data are expressed as means ± standard deviation. Statistical evaluation was carried out by one-way analysis of variance (ANOVA) followed by Dunnett's t-test for multiple comparisons. * = P < 0.05.

Using our previously described method we sought to determine the ability of isolates from the four DENV serotypes to prevent STAT1 phosphorylation after stimulation with IFN-β. To determine the relative percent inhibition of pSTAT by DENV strains compared to 16681 we calculated the difference between the expected pSTAT1 inhibition at the obtained percent of infection and the observed pSTAT1 inhibition. A phylogenetic analysis of the sequenced DENV strains from our Passive Dengue Surveillance System (PDSS) was performed and we selected those that represented ancestors of recently circulating viruses (S1A-S1D Fig. and S1 Table). A description of these viruses can be seen in Table 2 (see S2 Table for plaque sizes). Our results show that all of the DENV serotypes evaluated had the capacity to block the IFN-α/β response (Fig. 3A). However, there was some variability in the levels of inhibition of pSTAT1 by these DENV serotypes compared to 16681. DENV-1 & 2 had slightly lower levels of inhibition at-16% and-10% respectively. The lowest levels of inhibition were observed with DENV-3 at-34% and the highest levels were observed with DENV-4 at 10% (Fig. 3A). The largest (44%) difference in inhibition was observed between DENV-3 and-4.

**Table 2:** Description of DENV strains used in this study.

DENV Serotype	Genotype	Virus strain	Passage history^a^	Isolation / clinical diagnosis^b^	Location	Year
**DENV1**	American-African	101–001/PR1998	C-3	Human/D	Puerto Rico	1998
**DENV2**	Asian-American	BID-V681	C-3	Human/D	Puerto Rico	1998
**DENV3**	Indian Subcontinent	BID-V1610	C-3	Human/D	Puerto Rico	2004
**DENV4**	Indonesia	BID-V2442	C-4	Human/D	Puerto Rico	1998
**DENV2**	American	131	C-6	Human/D	Mexico	1992
**DENV2**	American	IQT2133	C-7	Human/D	Peru	1996
**DENV2**	American	PR159	high passage	Human/D	Puerto Rico	1959
**DENV2**	American	Ven2	C-12	Human/D	Venezuela	1987
**DENV2**	South East Asian	16681	C-4, MK2–1, C-6	Human/SD	Thailand	1964
**DENV2**	Southeast Asian	K0049	C-4	Human/SD	Thailand	1995
**DENV2**	Southeast Asian	TH/DB052/2003	C-5	Human / unknown	Thailand	2003
**DENV2**	Southeast Asian	203–001/TW1987	C-2	Human / unknown	Taiwan	1987
**DENV2**	Asian-American	Mara3	C-6	Human/D	Venezuela	1990
**DENV2**	Asian-American	201–001/PR2006	C-5	Human/D	Puerto Rico	2006
**DENV2**	Asian-American	BID-V585	C-3	Human/D	Puerto Rico	2006
**DENV2**	cosmopolitan	1349	M-1, C-7	Human / unknown	Burkina Faso	1982
**DENV2**	cosmopolitan	Ara6894	S-4, C-3	Mosquito	Burkina Faso	1986
**DENV2**	sylvatic	DakAr510	S-4, C-5	Mosquito	Ivory Coast	1980
**DENV2**	sylvatic	DakAr75505	A-5, C-4	Mosquito	Senegal	1991
**DENV2**	sylvatic	DkD811	C5	Human/SD	Malaysia	2008

Note. ^a^ A, AP61 mosquito cell line;C, C6/36 mosquito cell line; M, whole mosquito; MK2, Rhesus monkey kidney cells S, suckling mice.

^b^ D indicates dengue; SD indicates severe dengue

To explore the possibility of differences in IFN-α/β antagonism by DENV strains associated with different pathogenicities, we studied several strains from the clinically well-characterized genotypes of DENV serotype 2 (DENV-2). A549 cells were infected with strains from the American, Asian, American/Asian, Cosmopolitan, and sylvatic genotypes. As observed with the four DENV serotype strains examined, the levels of inhibition of pSTAT1 by most of the DENV-2 strains was similar to that observed with strain 16681. Most of the strains did not display a consistent relative increase or decrease in inhibition of pSTAT1 by genotype (Fig. 3B). Interestingly, the DENV-2 sylvatic genotype consistently displayed higher levels of pSTAT1 inhibition (33–61%) compared to 16681 and all other strains (p < 0.01). The examination of all of the DENV strains in this study suggests that all DENVs are capable of inhibiting pSTAT1 and that this function, albeit with small variations, is highly conserved.

### DENV replication rate does not correlate with IFN-α/β blocking ability

**Fig 4:**
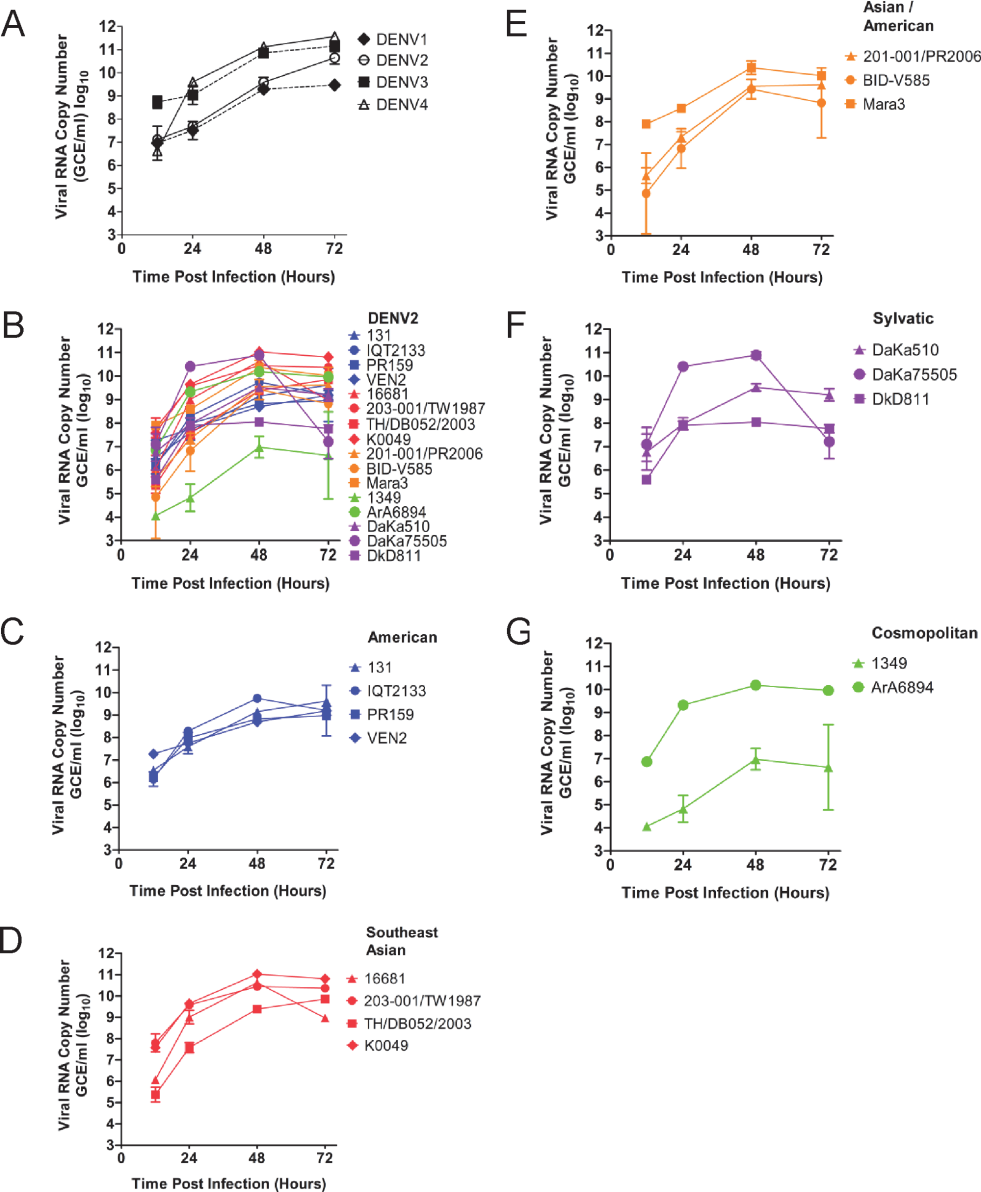
Comparison of DENV replication in A549 cells detected by RT-PCR. A549 cells were infected with representative DENV strains at an MOI = 0.1 for 1 hour. After washing, cells were incubated for the time period indicated (12, 24, 48, or 72 h) and cell supernatants were harvested for quantification of viral genomes by real-time RT-PCR as described in Materials and Methods. Experiments were performed in triplicate. Results shown are representative of two independent experiments.

The replication rate of all the DENVs used in this study was measured to determine if it could play a role in the observed IFN-α/β antagonism of these viruses. Only DENV-1 displayed lower growth than the other three DENV serotypes (Fig. 4A). However, the level of pSTAT1 inhibition of DENV-1 was not as low as DENV-3, which displayed a similar replication rate as DENV-4, the best pSTAT1 inhibitor. Measurement of the replication rate of DENV-2 strains showed that the Asian strains replicated to higher levels than American/Asian and American strains (Figs. 4B, 3C, 3D & 3E). The replications rates of strains in the Cosmopolitan and sylvatic genotypes were variable and inconsistent within the genotype (Fig. 4E & 4F). DENV DkD811 displayed one of the lowest replication rates. Together, the data demonstrates that the replication rate of DENVs does not cause interference in our model assessing the IFN inhibitory ability of these viruses.

### DENV sylvatic strains are unable to inhibit pSTAT1 in non-human primate cell lines

**Fig 5:**
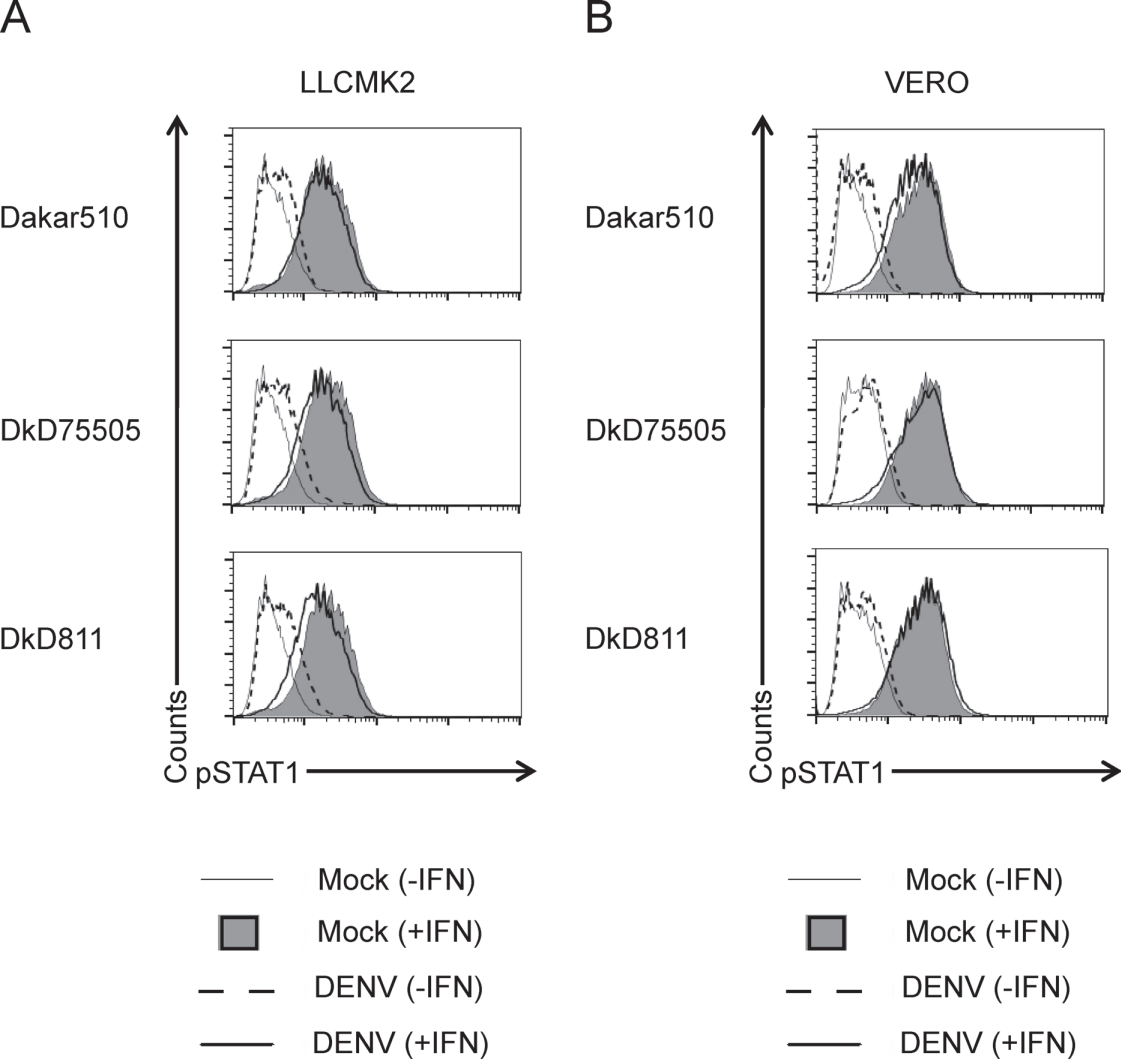
STAT1 is phosphorylated in non-human primate cell lines infected with sylvatic DENV and stimulated with IFN-β. Dengue sylvatic strains Daka510, DakAr 75505, and DKD811 were used to infect (**A**) LLCMK2 and (**B**) Vero cells at an MOI of 5 for 24 hours. Cells were then stimulated for 30 min. with IFN-β (500 U/ml) and co-stained with anti-pSTAT1 Alexa 647- and anti-dengue prM Alexa 488-conjugated antibodies. Cell fluorescence was measured on a BD FACS Calibur and data analysis was conducted using FlowJo software. Results shown are representative of two independent experiments.

It is hypothesized that sylvatic DENVs, mainly detected in West Africa and Malaysia, are transmitted in an enzootic cycle most likely between NHPs and arboreal *Aedes spp*. mosquitoes. Therefore, we wanted to determine whether the increased inhibition of STAT1 phosphorylation by sylvatic DENVs was also observed in NHP cells. Surprisingly, none of the sylvatic DENV strains used in this study were able to prevent STAT1 phosphorylation. Results were comparable in LLCMK2 (*Rhesus macaque*) and Vero (*Cercopithecus aethiops*) cell lines (Fig. 5).

### Inhibition pSTAT1 by DENV 16681 is minimal in non-human primate cell lines

**Fig 6:**
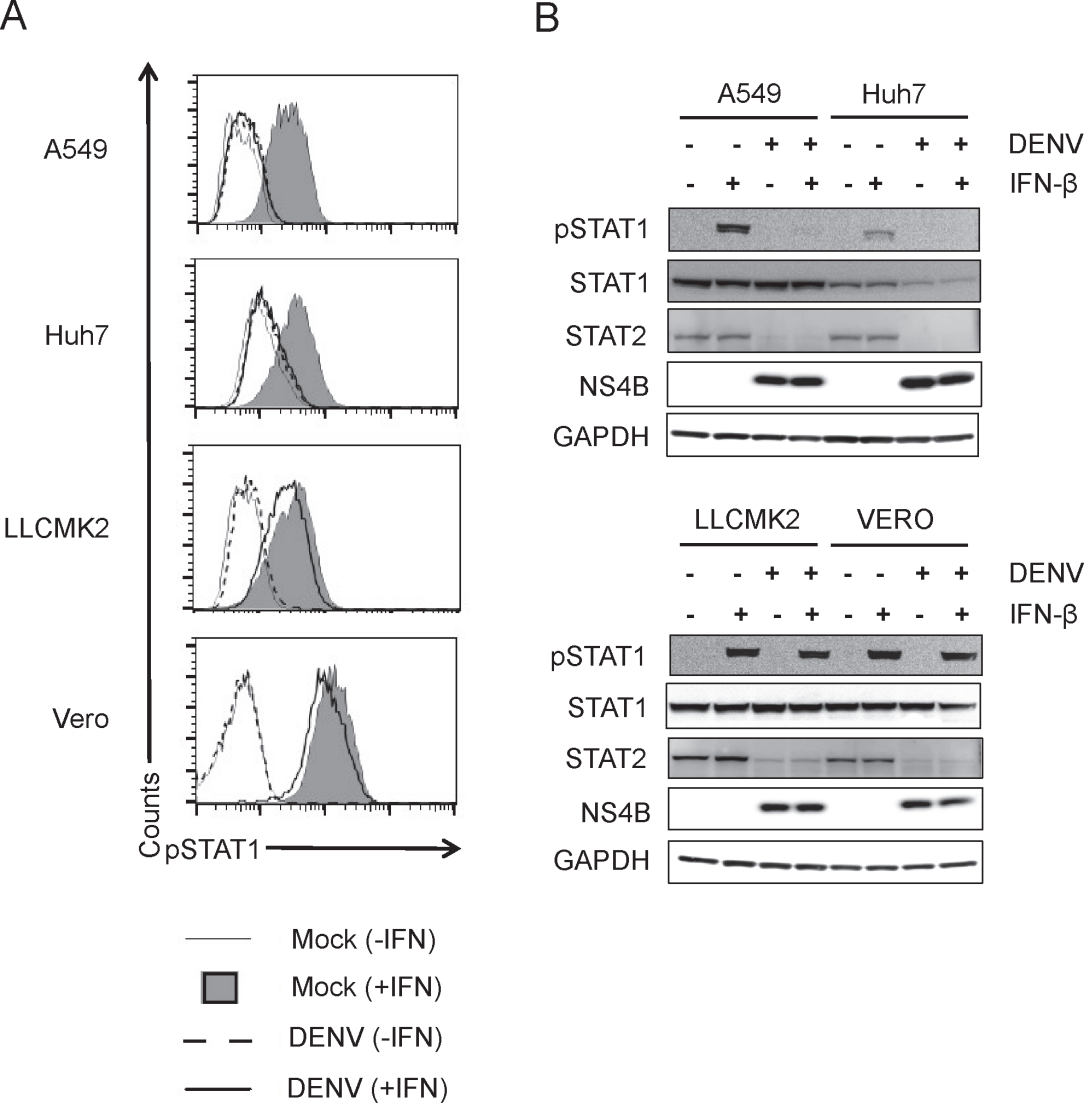
Inhibition of IFN-β signaling in DENV-2 16681 in human and non-human primate cell lines. DENV-2 16681 was used to infect human (A549 & Huh7) and NHP (LLCMK2 & Vero) cell lines at a MOI of 5 for 24 hours. Cells were then stimulated for 30 min. with IFN-β (500 U/ml) and processed for (**A**) flow cytometry of pSTAT1 or (**B**) Western blots of pSTAT1, STAT1, STAT2, NS4B and GAPDH. Results shown are representative of two independent experiments.

The unexpected finding that sylvatic DENVs were unable to inhibit STAT1 phosphorylation in NHP cell lines led us to question whether this observation was unique to these viruses. For this reason, we compared inhibition of IFN signaling by DENV-2 strain 16681 in both human (A549, Huh7) and NHP (LLCMK2, Vero) cell lines. Flow cytometry analysis of STAT1 phosphorylation in the DENV+ population revealed that there is a slight reduction in NHP cells compared to uninfected cells stimulated with IFN-β (Fig. 6A). As expected, DENV 16681 inhibited phosphorylation of STAT1 and caused STAT2 degradation in human cell lines. However, in NHP cell lines, STAT1 was phosphorylated in infected cells after IFN stimulation, but degradation of STAT2 was observed (Fig. 6A & B). This demonstrates a significantly reduced ability of DENVs to inhibit STAT1 phosphorylation in NHP cell lines.

### DENV can inhibit pSTAT1 in non-human primate myeloid dendritic cells and stimulates production of IFN in infected cells

**Fig 7:**
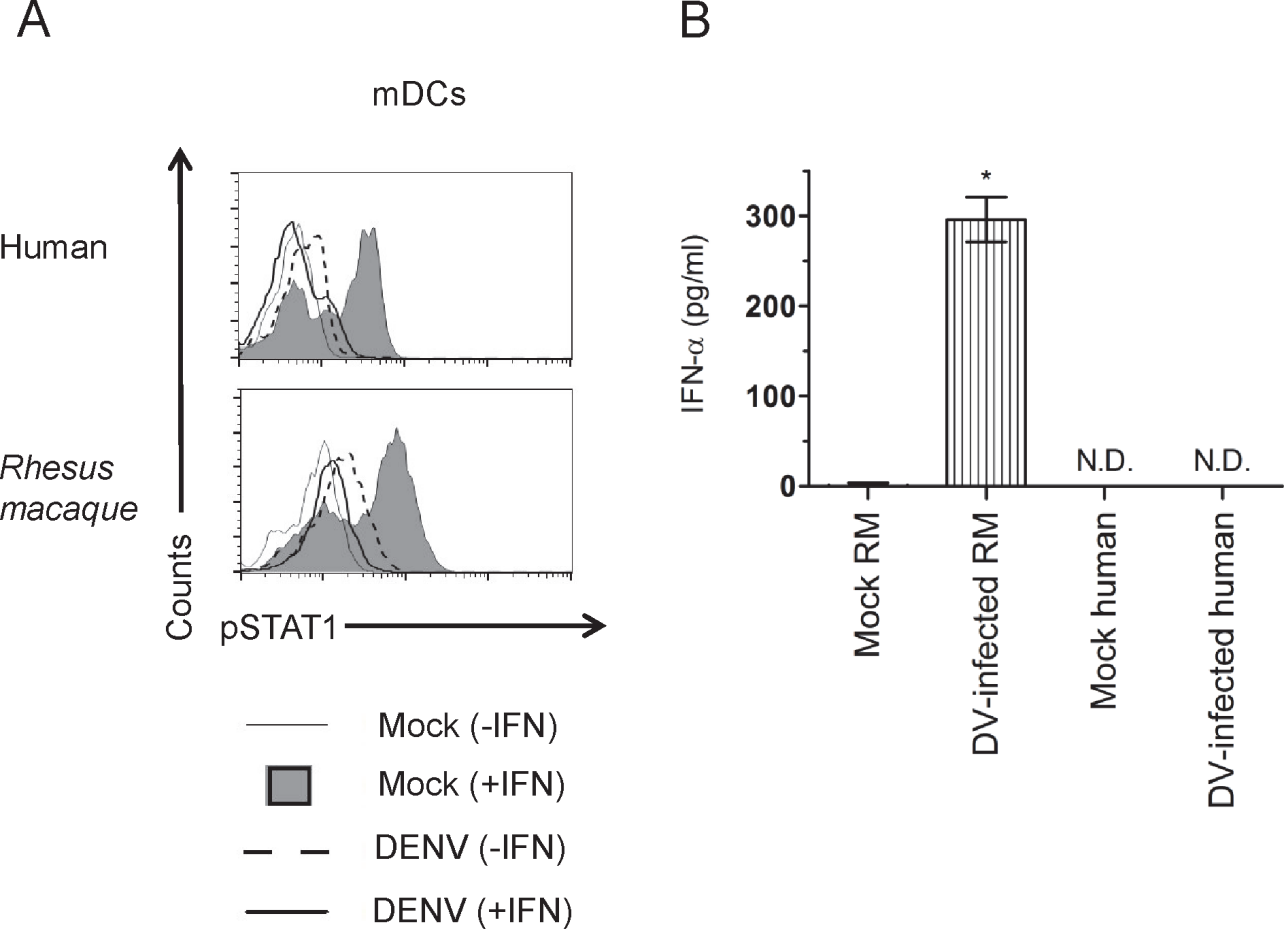
DENV2 16681 blocks STAT1 phosphorylation but can’t inhibit production of IFN-α in primary myeloid dendritic cells. Dendritic cells from representative were mock-infected or infected with DENV-1 16681 at an MOI of 5 for twenty-four hours. (A) Cells were stimulated for 30 min. with IFN-β (500 U/ml) and co-stained with anti-pSTAT1 Alexa 647- and anti-dengue prM Alexa 488-conjugated antibodies. Cell fluorescence was measured on a BD FACS Calibur and data analysis was conducted using FlowJo software. (B) The concentrations of IFN-α in the supernatants were quantified by ELISA. Results shown for flow cytometry are representative of two human and two Rhesus macaque samples. Results shown for IFN-α production were done in triplicate and represent the values obtained from two Rhesus macaques.

In order to determine if the observed reduction in pSTAT1 is also observed in primary cells we differentiated CD14+ monocytes into myeloid dendritic cells. Analysis of human dendritic cells infected with or without dengue and stimulated with IFN revealed results comparable to those obtained with human cell lines (A549, Huh7). Phosphorylation of STAT1 was observed in uninfected dendritic cells after stimulation with IFN. These levels of pSTAT1 did not increase in DENV infected human and *Rhesus macaque* dendritic cells after stimulation with IFN (Fig. 7A). An increase in pSTAT1 was observed in human and *Rhesus macaque* dendritic cells that were infected with DENV, but not stimulated with IFN, suggesting that IFN was being produced. Testing of *Rhesus macaque* dendritic cell supernatants twenty-four hours post-infection revealed the presence of IFN-α. No IFN-α was detected in human dendritic cell supernatants (Fig. 7B).

## Discussion

There is mounting evidence for conserved mechanisms among flaviviruses to counteract the IFN-α/β response [26–29,39]. However, some studies suggest that not all flaviviruses are capable of inhibiting STAT1 phosphorylation and in some instances it resulted in reduced pathogenicity [30–33]. Most of these comparative studies are limited in the number of virus strains analyzed and the clinical data available. Therefore we aimed to do a more exhaustive study evaluating the capacity of DENVs to interfere with IFN-α/β signaling and if any observed difference could correlate with disease outcome. A previous study on DENV-induced STAT2 degradation and our unpublished observations between DENV-2 strains shows that this inhibitory mechanism is conserved among all DENVs [40]. Therefore, we focused on phosphorylation of STAT1 as a marker for differences in IFN-α/β signaling antagonism by DENVs.

Signaling though the Jak/Stat pathway can occur through STAT1 and STAT2 homodimers or heterodimers. The presence of both molecules is not required for activation of the Jak/Stat pathway to occur. However, the ISG response is more potent when both molecules are present. This general observation has been studied in the context of dengue virus as well. Studies by Shresta et al. demonstrated that a STAT1-independent IFN response confers protection from DENV infection in mice [41]. This suggested that signaling through STAT2 homodimers could mount an antiviral response that was sufficient to combat DENV infection. Furthermore, studies by Perry et. al. showed that both STAT-1 and STAT-2 single-deficient mice can survive a DENV challenge whereas STAT1/2 double deficient mice succumbed to early death [42]. This showed that STAT1 homodimers could also mount an antiviral response that was sufficient to combat DENV infection. These results suggest that there is a compensatory mechanism that protects against DENV in the absence of either one of the STAT proteins. Additional experiments done by Perry et. al suggest that STAT1 plays a larger role in the anti-DENV response than STAT2. Part of this evidence is supported by the fact that the viral load in STAT2 deficient mice was lower than their STAT1 counterparts at multiple time points after infection [42]. Together these data suggest that inactivation of either STAT1 or STAT2 alone is not sufficient to eliminate the antiviral response.

Studies evaluating differences in the cellular response to flaviviruses frequently encounter the difficulty of performing an accurate comparative analysis between strains. DENV isolates from humans or mosquitoes can differ in their morphology, plaque size and replication rate [43–45]. This makes it difficult to normalize for infectivity when comparisons are made between virus strains. For some DENVs, viral quantification by plaque assay can be difficult. Viral plaques can sometimes be very small and/or difficult to discern. For this reason, we developed a method akin to an ELISA assay for quantifying antagonism of IFN-α/β signaling based on the measurement of pSTAT1 inhibition in DENV(+) cells using flow cytometry. Comparing inhibition of IFN-α/β signaling molecules between DENV strains by RT-PCR, luciferase assays and Western blot analysis in infected cells can lead to misleading results since uninfected cells will be a confounding factor in the results. The use of Western blot analysis can also be problematic when MAbs to variable regions or polyclonal antibodies are used as a control for virus proteins. In these cases, even when plaque size and infectivity are same, the virus band signal intensity is greater in viruses that are more similar to the virus used for antibody development. Our data shows that when the percent of infected cells is not taken into account it can lead to an over- or underestimation of the virus’s antagonistic activity. The measurement of pSTAT1 in the DENV(+) population without standardization was inadequate for a comparative analysis between strains because the level of pSTAT1 inhibition increases with the percentage of infected cells. The virus replication rate did not have a confounding effect in our analysis as it did not correlate with the ability of DENVs to block phosphorylation of STAT1.

To our knowledge, we present the first concurrent comparison of all four DENV serotypes for their ability to block IFN-α/β signaling. All of the representative clinical isolates representing the four DENV serotypes were capable of inhibiting STAT1 phosphorylation to a significant degree, with low variability. Prospective clinical studies will be needed to determine whether this moderate reduction in inhibition of IFN-α/β signaling by DENV-3 results in pathogenic differences between DENV serotypes or strains. DENV-2 viruses, which have been better described clinically as to their pathogenic potential than other DENV serotypes, did not vary greatly in their ability to inhibit pSTAT1 [4,7]. Although DENV-2 Asian strains have been shown to cause more severe disease compared to American strains, their ability to inhibit pSTAT1 did not differ significantly from the other genotypes.

Viruses from the DENV-2 sylvatic genotype distinguished themselves by consistently displaying an increased capacity to inhibit STAT1 phosphorylation. The replication rate did not have a confounding effect in our analysis as they did not correlate with the ability of DENVs to block phosphorylation of STAT1. Our findings are consistent with previous observations showing that the DENV-2 Asian genotype has a similar level of replication, but attains higher virus titers than all other genotypes. Therefore, the increased peak viral load observed in strains of the DENV-2 Asian genotype are likely capable of causing an overall increased capacity to inhibit IFN-α/β signaling and induce more severe illness than other genotypes. This is simply due to the fact of increased presence of the virus and not due to a viral genetic determinant conferring increased virulence because of an improved capacity to antagonize IFN-α/β.

Documentation of human cases with viruses of the sylvatic genotype is not common, but spillover events have been observed in West Africa and Malaysia [46–49]. The cases described in West Africa are mostly of mild disease. Only two cases of dengue hemorrhagic fever caused by sylvatic dengue serotype 2 viruses have been documented. One occurred in West Africa and the other in Malaysia [47,50]. Therefore, it is difficult for us to address the possible implications this increased inhibition could have on clinical outcomes of human infections. Sylvatic DENV strains are considered ancestors of the current circulating genotypes that infect humans and are maintained through a mosquito-NHP cycle. Although we are not certain whether the lack of inhibition of pSTAT1 by DENVs occurs in non-hematopoietic primary NHP cells as observed in NHP cell lines, we did observe an increased ability of sylvatic strains to inhibit pSTAT in human cell lines. Inhibition of pSTAT by DENV remained intact in primary Rhesus macaque dendritic cells. One limitation of our study is that we were unable to assess expression of ISGs due to lack of expression of antiviral proteins in the NHP cell lines. The lone study to date that utilized a sylvatic DENV strain to infect NHPs showed that the viremia was similar to that of human-endemic DENV-2 but lasted for a shorter period of time and no differences in pathogenicity were observed [51]. Although the sample size was small in this study, it suggests that the increased ability of sylvatic strains to inhibit pSTAT1 does not result in any obvious pathogenic differences in NHPs when compared to infections by human-endemic DENVs.

We observed production of IFN-α in DENV-infected primary myeloid Rhesus macaque dendritic cells that had not been stimulated. In contrast, no production of IFN-α was detected from DENV-infected primary myeloid human dendritic cells. Our observations in human dendritic cells are in concordance with those obtained by Rodriguez-Madoz et. al. under similar experimental conditions in which they also used DENV-2 16681 in their study and did not observe production of IFN-α/β at 24 hours post-infection with different MOIs ranging from 0.2–25. These observation together with the high levels of IFN-α detected in NHP dendritic cell supernatants suggests that inhibition of IFN-α production by DENV in NHPs does not occur. These results highlight a key difference in the IFN response between humans and NHPs in primary cells.

These limited, but interesting observed differences between human and NHPs cells deserve further study as they may add to the growing body of evidence to the distinct immune responses in primate hosts. It is known that humans are more severely affected than NHPs to a large number of diseases. Examples include HIV infections progressing to AIDS, *Plasmodium falciparum* infection progressing to malaria, and adverse complications following infections with hepatitis B, C and DENV [52,53]. The differences in susceptibility are thought to arise from inter-species differences in the immune response to infections. For example, in sooty mangabey monkeys which are unaffected by SIV or Yellow Fever, the innate and adaptive T cell proliferative responses are limited compared to *Rhesus macaques* and humans [54,55]. In a comparison of genome-wide gene expression levels between humans, chimpanzees, and rhesus macaques, it was observed that the innate response associated with viral infections is often lineage-specific [56]. Moreover, even though expression of PAMPs is similar in human and *Rhesus macaque* myeloid dendritic cells, the cytokine response differs [57]. This suggests that although humans and NHPs have similar innate immune signaling pathways, there are differences that will result in different outcomes in terms of expression and pathogenic outcome.

Studies by Umareddy et. al [30] suggested that some DENV strains are unable to suppress pSTAT1. Among these was TSV01, a cosmopolitan strain with 98.9% amino acid similarity to 1349 that was used in our experiments. Our results showed that 1349 was able to inhibit pSTAT1 at a level relatively higher than strain 16681. Our comparison of the NS4B amino acid sequence between TSV01 and 1349 shows that they are identical. These results suggest that either NS4B from TSV01 should be capable of inhibiting pSTAT1 or that another dengue protein aside from NS4B plays a more prominent role in preventing STAT1 phosphorylation in these strains.

The use of targeted mutagenesis of IFN-α/β-antagonizing flavivirus proteins to make IFN-α/β-sensitive viruses has been proposed as a method of vaccine attenuation [32]. Our results suggest that contrary to what has been published for other DENVs and flaviviruses, most, if not all DENVs have the capacity to inhibit STAT1 phosphorylation. This suggests that the selection of naturally occurring IFN-α/β-sensitive DENVs is not likely and attenuation must be achieved through genetic means. Targeted sites should be chosen carefully when designing DENV vaccine candidates and sensitivity to IFN-α/β evaluated through a rigorous method such as the one we have presented here.

## Supporting Information

S1 Fig (A-D) Maximum likelihood phylogeny of DENV strains. The unrooted maximum likelihood phylogenetic trees were constructed based on envelope gene sequences from serotypes of: S1A, DENV-1; S1B, DENV-2; S1C, DENV-3; and S1D, DENV-4. Virus strains utilized in experiments for this study are indicated with a black dot (•). Bootstrap values based on 1000 replicas are shown at each main branch.
(TIF)

S1 Table Taxa labels, strain names, and GenBank accession numbers for DENVs used to construct phylogenetic trees for Supplemental Figures S1A-D Fig. Strains used in this study are highlighted in grey.
(DOCX)

S2 Table Plaque sizes of representative DENV strains. Clinical isolates from each DENV (1–4) serotype or from all DENV-2 genotypes (American, Southeast Asian, Asian/American, and cosmopolitan).
(DOCX)
