# Supplementary figures and images for: Differences in Type I Interferon Signaling Antagonism by Dengue Viruses in Human and Non-Human Primate Cell Lines
Source: PLoS Negl Trop Dis. 2015 Mar 13;9(3):e0003468. doi: 10.1371/journal.pntd.0003468 (PMC4359095; doi:10.1371/journal.pntd.0003468)

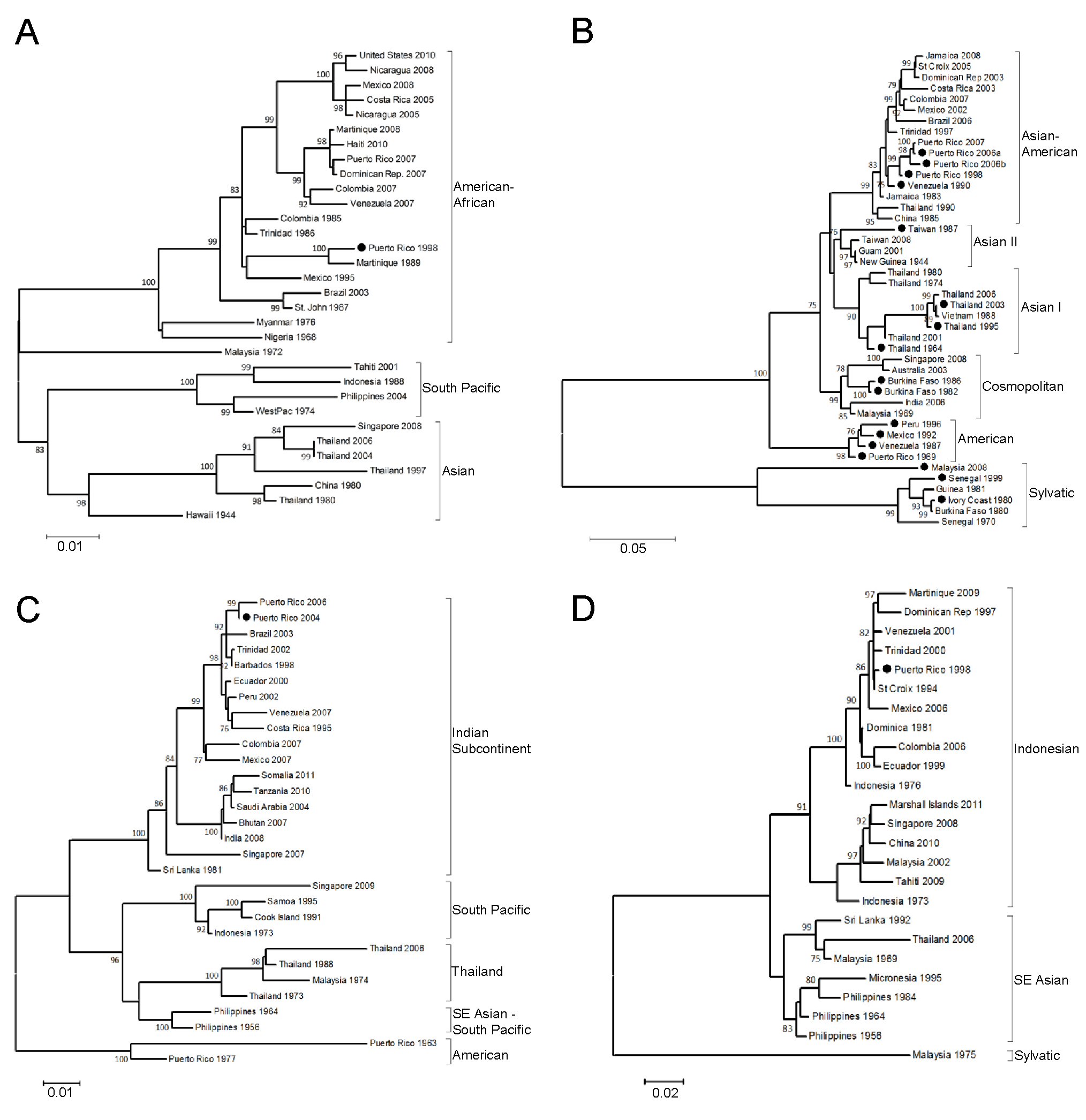

Supplement: S1 Fig — The unrooted maximum likelihood phylogenetic trees were constructed based on envelope gene sequences from serotypes of: S1A, DENV-1; S1B, DENV-2; S1C, DENV-3; and S1D, DENV-4. Virus strains utilized in experiments for this study are indicated with a black dot (•). Bootstrap values based on 1000 replicas are shown at each main branch. (TIF) [file pntd.0003468.s001.tif]
