# Supplementary material for: Differences in Type I Interferon Signaling Antagonism by Dengue Viruses in Human and Non-Human Primate Cell Lines
Source: PLoS Negl Trop Dis. 2015 Mar 13;9(3):e0003468. doi: 10.1371/journal.pntd.0003468 (PMC4359095; doi:10.1371/journal.pntd.0003468)
Supplement: S1 Table — Strains used in this study are highlighted in grey. (DOCX) [file pntd.0003468.s002.docx]

Table S1

| **Serotype** | **Taxa label** | **Strain** | **GenBank accession number** |
| --- | --- | --- | --- |
| 1 | Brazil 2003 | BR/DB001/2003 | JF804014 |
| 1 | China 1980 | GZ-China 80 | AF350498 |
| 1 | Colombia 1985 | 347869 | AF425616 |
| 1 | Colombia 2007 | CI/DB002/2007 | JF804015 |
| 1 | Costa Rica 2005 | CR/DB003/2005 | JF804016 |
| 1 | Dominican Rep. 2007 | DR/DB004/2007 | JF804017 |
| 1 | Haiti 2010 | HA/DB067/2010 | JF969282 |
| 1 | Hawaii 1944 | HAW44 | AB609588 |
| 1 | Indonesia 1988 | A88 | AB074761 |
| 1 | Malaysia 1972 | P72-1244 | AF425622 |
| 1 | Martinique 1989 | MQ/DB005/1989 | JF804018 |
| 1 | Mexico 1995 | 4942/QuintanaRoo | DQ341194 |
| 1 | Mexico 2008 | BID-V3677 | GU131965 |
| 1 | Myanmar 1976 | 228686 | AF425615 |
| 1 | Nicaragua 2005 | BID-V536 | FJ850113 |
| 1 | Nicaragua 2008 | BID-V2646 | GQ199858 |
| 1 | Nigeria 1968 | 28328 | AF425625 |
| 1 | Philippines 2004 | PH/DB007/2004 | JF804020 |
| 1 | Puerto Rico 1998 | 101-001/PR1998 | KC812277 |
| 1 | Singapore 2008 | EHI0266 | JN022600 |
| 1 | St. John 1987 | SJ/DB010/1987 | JF804023 |
| 1 | Tahiti 2001 | TI/DB011/2001 | JF804024 |
| 1 | Thailand 2006 | TH/DB047/2006 | JF812097 |
| 1 | Thailand 1980 | PUO359 | AF425630 |
| 1 | Thailand 2004 | TH/DB050/2004 | JF812100 |
| 1 | Thailand 1997 | TH/DB012/1997 | JF804025 |
| 1 | Trinidad 1986 | 86471 | AF425639 |
| 1 | United States 2010 | US/DB081/2010 | JQ425070 |
| 1 | Venezuela 1997 | 28164 | AF425634 |
| 1 | Venezuela 2007 | VE/DB013/2007 | JF804026 |
| 1 | WestPac 1974 | WestPac/NIBSC | M23027 |
| 2 | Australia 2003 | TSV01 | AY037116 |
| 2 | Brazil 2006 | BR/DB015/2006 | JF804028 |
| 2 | Burkina Faso 1980 | Dak Ar A2022 | EF105386 |
| 2 | Burkina Faso 1982 | 1349 | EU056810 |
| 2 | Burkina Faso 1986 | Ara6894 | HM234642 |
| 2 | China 1985 | isolate 04 | AF119661 |
| 2 | Colombia 2007 | CI/DB016/2007 | JF804029 |
| 2 | Costa Rica 2003 | CR/DB017/2003 | JF804030 |
| 2 | Dominican Rep. 2003 | DR/DB018/2003 | JF804031 |
| 2 | Guam 2001 | GU/DB019/2001 | JF804032 |
| 2 | Guinea 1981 | PM33974 | EF105378 |
| 2 | India 2006 | IN/DB020/2006 | JF804033 |
| 2 | Ivory Coast 1980 | Dak Ar510 | EF105381 |
| 2 | Jamaica 1983 | 1408 | D45392 |
| **Serotype** | **Taxa label** | **Strain** | **GenBank accession number** |
| 2 | Jamaica 2008 | JA/DB021/2008 | JF804034 |
| 2 | Malaysia 1969 | P7-863 | AF231716 |
| 2 | Malaysia 2008 | DkD811 | FJ467493 |
| 2 | Mexico 1992 | 131 | FJ467493 |
| 2 | Mexico 2002 | MX/DB022/2002 | AY158332 |
| 2 | Peru 1996 | IQT2133 | AY577439 |
| 2 | Puerto Rico 1969 | PR159 | L0046 |
| 2 | Puerto Rico 1998 | BID-V681 | EU482738 |
| 2 | Puerto Rico 2006a | 201-001/PR2006 | KC812279 |
| 2 | Puerto Rico 2006b | BID-V585 | EU529706 |
| 2 | Puerto Rico 2007 | PR/DB023/2007 | JF804036 |
| 2 | Senegal 1970 | Dak HD10674 | EF105384 |
| 2 | Senegal 1999 | Dak Ar75505 | EF457904 |
| 2 | Singapore 2008 | EHI1158 | JN030200 |
| 2 | St Croix 2005 | SC/DB024/2005 | JF804037 |
| 2 | Taiwan 1987 | 203-001/TW1987 | KC812278 |
| 2 | Taiwan 2008 | BID-V5056 | HQ891024 |
| 2 | Thailand 1964 | 16681 | M24447 |
| 2 | Thailand 1974 | S16803 | GU289914 |
| 2 | New Guinea 1944 | New Guinea C | AF038403 |
| 2 | Thailand 1980 | PUO312 | AF264053 |
| 2 | Thailand 1990 | Th0284 | DQ181801 |
| 2 | Thailand 1995 | K0049 | AY185337 |
| 2 | Thailand 2001 | TH/DB064/2001 | JF812114 |
| 2 | Thailand 2003 | TH/DB052/2003 | JF812102 |
| 2 | Thailand 2006 | TH/DB061/2006 | JF812111 |
| 2 | Trinidad 1997 | TR/DB025/1997 | JF804038 |
| 2 | Venezuela 1987 | Ven2 | AY158328 |
| 2 | Venezuela 1990 | Mara3 | AY158329 |
| 2 | Vietnam 1988 | VN/DB014/1988 | JF804027 |
| 3 | Barbados 1998 | BA/DB027/1998 | JF804040 |
| 3 | Bhutan 2007 | SV0786 | FJ606712 |
| 3 | Brazil 2003 | BR/DB028/2003 | JF804041 |
| 3 | Colombia 2007 | CI/DB029/2007 | JF804042 |
| 3 | Cook Island 1991 | CK/DB030/1991 | JF804043 |
| 3 | Costa Rica 1995 | CR/DB026/1995 | JF804039 |
| 3 | Ecuador 2000 | EC/DB032/2000 | JF804045 |
| 3 | India 2008 | DEL-72 | GQ466079 |
| 3 | Indonesia 1973 | 228761 | L11425 |
| 3 | Malaysia 1974 | 1300 | L11429 |
| 3 | Mexico 2007 | MX/DB031/2007 | JF804046 |
| 3 | Peru 2002 | PE/DB034/2002 | JF804047 |
| 3 | Philippines 1956 | H87 | AB609590 |
| 3 | Philippines 1964 | S16562 | JF812106 |
| 3 | Puerto Rico 1963 | PR6 | L11433 |
| 3 | Puerto Rico 1977 | 1340 | L11434 |
| **Serotype** | **Taxa label** | **Strain** | **GenBank accession number** |
| 3 | Puerto Rico 2004 | BID-V1610 | FJ024469 |
| 3 | Puerto Rico 2006 | PR/DB035/2006 | JF804048 |
| 3 | Samoa 1995 | SA/DB036/1995 | JF804049 |
| 3 | Saudi Arabia 2004 | 6805 | AM746229 |
| 3 | Singapore 2007 | EHI0235 | JN022604 |
| 3 | Singapore 2009 | EHI0040 | JN030162 |
| 3 | Somalia 2011 | SO/DB131/2011 | KC848589 |
| 3 | Sri Lanka 1981 | 1326 | L11431 |
| 3 | Tanzania 2010 | Hu/Tanzania/08/2010 | AB549332 |
| 3 | Thailand 1973 | CH53489 | DQ863638 |
| 3 | Thailand 1988 | PaH881/88 | AF349753 |
| 3 | Thailand 2006 | TH/DB055/2006 | JF812105 |
| 3 | Trinidad 2002 | TR/DB037/2002 | JF804050 |
| 3 | Venezuela 2007 | VE/DB031/2007 | JF804044 |
| 4 | China 2010 | D10168-GZ | JN029829 |
| 4 | Colombia 2006 | CI/DB039/2006 | JF804052 |
| 4 | Dominica 1981 | 814669 | AF326573 |
| 4 | Dominican Rep 1997 | DR/DB040/1997 | JF804053 |
| 4 | Ecuador 1999 | EC/DB041/1999 | JF804054 |
| 4 | Indonesia 1973 | 30153 | U18428 |
| 4 | Indonesia 1976 | S1036 | U18429 |
| 4 | Malaysia 1969 | P7-1006 | AF231722 |
| 4 | Malaysia 1975 | P75-514 | AF231723 |
| 4 | Malaysia 2002 | SB8572 | FM986674 |
| 4 | Marshall Islands 2011 | RMI/DB104/2011 | JX891655 |
| 4 | Martinique 2009 | 490930077 | JN022606 |
| 4 | Mexico 2006 | MX/DB042/2006 | JF804055 |
| 4 | Micronesia 1995 | MC/DB043/1995 | JF804056 |
| 4 | Philippines 1956 | H241 | AB609591 |
| 4 | Philippines 1964 | 16589 | U18434 |
| 4 | Philippines 1984 | 12123 | U18435 |
| 4 | Puerto Rico 1998 | BID-V2442 | FJ882596 |
| 4 | Singapore 2008 | EHI2641 | HQ875339 |
| 4 | Sri Lanka 1992 | UNC4009 | U18437 |
| 4 | St Croix 1994 | SC/DB045/1994 | JF804058 |
| 4 | Tahiti 2009 | PF09/270409-182 | JN832530 |
| 4 | Thailand 2006 | TH/DB057/2006 | JF812107 |
| 4 | Trinidad 2000 | TR/DB046/2000 | JF804059 |
| 4 | Venezuela 2001 | VE/DB038/2001 | JF804051 |
