# Supplementary material for: Differences in Type I Interferon Signaling Antagonism by Dengue Viruses in Human and Non-Human Primate Cell Lines
Source: PLoS Negl Trop Dis. 2015 Mar 13;9(3):e0003468. doi: 10.1371/journal.pntd.0003468 (PMC4359095; doi:10.1371/journal.pntd.0003468)
Supplement: S2 Table — Clinical isolates from each DENV (1–4) serotype or from all DENV-2 genotypes (American, Southeast Asian, Asian/American, and cosmopolitan). (DOCX) [file pntd.0003468.s003.docx]

Table S2

| **SEROTYPE** | **GENOTYPE** | **STRAIN** | **PLAQUE SIZE (avg. mm ± s.d.)** |
| --- | --- | --- | --- |
| DENV1 | American-African | 101-001/PR1998 | 4.35 **±** 1.66 |
| DENV2 | Asian-American | BID-V681 | 3.62 **±** 1.04 |
| DENV3 | Indian Subcontinent | BID-V1610 | 5.14 **±** 1.02 |
| DENV4 | Indonesia | BID-V2442 | 5.53 **±** 2.19 |
| DENV2 | American | 131 | 1.89 **±** 0.7 |
| DENV2 | American | IQT2133 | 3.5 **±** 0.67 |
| DENV2 | American | PR159 | 1.75 **±** 0.32 |
| DENV2 | American | Ven2 | 0.65 **±** 0.14 |
| DENV2 | Asian | 16681 | 3.37 **±** 0.75 |
| DENV2 | Asian | K0049 | 3.8 **±** 1.41 |
| DENV2 | Asian | TH/DB052/2003 | 6.2 **±** 1.71 |
| DENV2 | Asian | 203-001/TW1987 | 3.36 **±** 0.78 |
| DENV2 | Asian-American | Mara3 | 0.79 **±** 0.19 |
| DENV2 | Asian-American | 201-001/PR2006 | 2.52 **±** 0.71 |
| DENV2 | Asian-American | BID-V585 | 4.04 **±** 0.66 |
| DENV2 | cosmopolitan | 1349 | 3.57 **±** 0.65 |
| DENV2 | cosmopolitan | ArA6894 | 1.22 **±** 0.3 |
| DENV2 | sylvatic | Daka510 | 3.44 **±** 0.67 |
| DENV2 | sylvatic | DakAr75505 | 5.49 **±** 2.34 |
| DENV2 | sylvatic | DkD811 | 3.68 **±** 0.75 |
